# Induction of Migration and Collagen Synthesis in Human Gingival Fibroblasts Using Periodontal Ligament Stem Cell Conditioned Medium

**DOI:** 10.1055/s-0043-1764422

**Published:** 2023-04-27

**Authors:** Akkapol Banlue, Jirattikarn Kaewmuangmoon, Kajohnkiart Janebodin, Kallapat Tansriratanawong

**Affiliations:** 1 Department of Oral Medicine and Periodontology, Faculty of Dentistry, Mahidol University, Bangkok, Thailand; 2 Department of Anatomy, Faculty of Dentistry, Mahidol University, Bangkok, Thailand

**Keywords:** cell migration, collagen synthesis, culture media, conditioned, fibroblasts, periodontal ligament, stem cells

## Abstract

**Objective** This study aimed to examine the effect of periodontal ligament stem cell conditioned medium (PDLSC-CM) on human gingival fibroblast (HGF) migration and collagen synthesis.

**Materials and Methods** To assess cell viability, we extracted PDLSC-CM, and the total derived protein concentration was adjusted to 12.5 to 200 µg/mL, followed by treatment with HGFs. The viability of HGFs was observed for 24 hours using the MTT assay. Cell migration was monitored for 24 to 48 hours by wound healing and Boyden chamber assays. Collagen synthesis from HGFs was examined by picrosirius red dye and real-time polymerase chain reaction (PCR) to measure collagen type I and III gene expression for 7 to 10 days. A comparison among the groups was assessed using a one-way analysis of variance (ANOVA) and Bonferroni post hoc test, with the exception of the cell viability assay, which was subjected to Welch's test and Dunnett's T3 post hoc test.

**Results** HGF viability was significantly enhanced by 12.5, 25, and 50 µg/mL PDLSC-CM. The HGFs treated with 50 µg/mL PDLSC-CM promoted cell migration as shown by wound healing and Boyden chamber assays. At this concentration, collagen synthesis increased at 10 days. Collagen type I gene expression increased by 1.6-fold ( *p* < 0.001) and 4.96-fold ( *p* < 0.001) at 7 and 10 days, respectively. Collagen type III gene expression showed an increase of 1.76-fold ( *p* < 0.001) and 6.67-fold ( *p* < 0.001) at the same time points.

**Conclusion** Our study suggested that a low concentration of PDLSC-CM at 50 µg/mL has given an amelioration of HGFs providing for periodontal wound healing and periodontal regeneration, particularly migration and collagen synthesis.

## Introduction

Mesenchymal stem cells (MSCs) have shown promise for tissue regeneration in various organs, including periodontal defects, due to their biological potentials, such as self-renewal, multilineage differentiation, and immunomodulation. 1 Periodontal wound healing has been shown to be improved by MSCs derived from bone marrow as well as dental tissues. 2 One good MSC candidate from dental tissue sources for periodontal regeneration is periodontal ligament stem cells (PDLSCs). PDLSCs that express MSC markers can enhance periodontal regeneration. 3 PDLSCs have a high proliferation rate and can form mineralized tissues with the host's cementum and alveolar bone without causing tissue rejection. 4 To form a dental functional unit, PDLSCs generate collagen type I of periodontal ligamentlike structures to form Sharpey's fibers. For PDLSC paracrine function, it would be interesting to determine how secreted proteins from this stem cell type impact oral tissue healing.

Since direct transplantation of MSCs has several limitations, the conditioned medium (CM) is used instead of cell therapy. The CM is prepared in a laboratory by culturing stem cells in a serum-free medium for a period of time. Stem cell–derived CM could maintain tissue homeostasis via secretome release to the milieu. 5 The secretomes are a mixture of biological molecules, including lipids, proteins, nucleic acid, trophic factors such as extracellular vesicles (EVs), growth factors, and cytokines, which are necessary for reconstructive tissues. 6 Thus, utilizing CM may have biological effects on the cells. It can be accurately measured for proper dosages and produced in large quantities with a noninvasive extraction procedure, which is both time- and cost-saving. Therefore, utilizing a stem cell-derived CM may be useful in periodontal wound healing. According to Nagata and colleagues, periodontal ligament stem cell conditioned medium (PDLSC-CM) contains serine protease inhibitor E1, monocyte chemotactic protein-1, insulinlike growth factor (IGF) binding protein-2 and 6, and platelet-derived growth factor (PDGF), which enhance wound healing in animal models. Additionally, the PDLSC-CM group has a lower inflammatory response. 7 These cytokines released from the CM also promoted periodontal structure formation and Sharpey's fiber insertion. Qiu and colleagues confirmed that PDLSC-CM regenerated functional periodontal tissue. PDLSC-CM had immunomodulatory properties and increased osteogenesis markers. 8 Moreover, PDLSC-CM enhances osteoblast proliferation and osteoblastic differentiation marker expression. 9 It is owing to the fact that mesenchymal stem cell extracellular vesicles (MSC-EVs) play a fundamental role in various biological processes such as cell migration, proliferation, differentiation, angiogenesis, and immunomodulation by regulating cell-to-cell and cell-to-extracellular matrix interactions. 10 MSC-EVs also regulate the balance of the microenvironment due to tissue disturbance. 5 10 MSC-EVs promote bone and periodontal regeneration. 11

Human gingival fibroblasts (HGFs) are abundant in gingival connective tissue and play an essential role in soft-tissue repair and regeneration. In periodontal healing, HGFs are key cells in the proliferative and remodeling phases. HGFs secrete collagen types III and I in the early and late phases of wound healing, respectively. 12 Collagen type I, which is mainly found in gingival connective tissue, is necessary for promoting wound repair and hard tissue regeneration, including bone, dentin, and cementum. Collagen type III plays an important role in enhancing cell adhesion and cell proliferation and promotes elastic angiogenesis in wound contraction of the soft tissues. 13 However, the increase in ages is a major risk factor that can delay the healing of wound. 14 A previous study has demonstrated that aged mice had insufficient gingival fibroblast proliferation, migration, and collagen synthesis. 15 In addition, the aged mice had a lower angiogenesis than the young mice. 16 17 Systemic diseases or certain medications can hamper wound healing. Diabetic patients or patients intaking corticosteroids have delayed wound healing. 18 Interestingly, mesenchymal stem cell conditioned medium (MSC-CM) increases the proliferative rate and collagen synthesis of HGFs. 19 Therefore, the CM from PDLSC may have several advantages in periodontal healing and enhancing the ability of HGFs.

Nevertheless, there is a limitation in the data that describe the impacts of PDLSC-CM on gingival healing. Especially, the emphasis on how the PDLSC secretome affects the behavior of HGFs is noteworthy. Thus, enhancing the biological properties of HGFs may be a promising approach to promoting rapid wound healing. This research proposes to examine the potency of PDLSC-CM on HGF viability, migration, and collagen synthesis in vitro.

## Materials and Method

### Human Gingival Fibroblast Cell Culture

The HGF cell (ATCC number: CRL-2014, United States) was cultured in a complete medium containing Dulbecco's modified Eagle's medium (DMEM, Gibco, Thermo Fisher Scientific, Waltham, MA, United States), 10% fetal bovine serum (FBS; Merck, Sigma-Aldrich, St. Louis, MO, United States), and 1% antibiotic–antimycotic (Merck). These cells were incubated in 5% CO _2_ at 37°C. HGFs from passages 6 to 10 were used for the experiments.

### Conditioned Medium Collection and Preparation

PDLSC in this research comes from the human periodontal ligament fibroblast (HPDLF; Catalog Number: 2630, ScienCell, Carlsbad, CA, United States), which was characterized by stem cell markers from a previously report. 20 HPDLF from passages 5 to 8 were used for the experiments. PDLSC was cultured in DMEM with 10% FBS. For collection of CM from PDLSC, DMEM was replaced with a serum-free medium. Before a serum-free medium was added, the cells were rinsed twice with phosphate-buffered saline (PBS; Gibco). The cells were continuously cultured for 2 days and then CM was collected. CM was stored at –20°C until further use. The Bradford assay was performed to determine the total protein concentration in the CM. Bovine serum albumin (BSA; Bio-Rad, Hercules, CA, United States) was used as a standard for the relative protein concentration.

### Cell Viability Assay

The HGFs were seeded in a 96-well plate at a concentration of 1 × 10 ^4^ cells/well and incubated at 5% CO _2_ and 37°C for 24 hours. Then, the HGFs were replaced with various concentrations of PDLSC-CM using a twofold dilution technique in serum-free DMEM, including 12.5, 25, 50, 100, and 200 µg/mL. HGFs treated with serum-free DMEM were used as controls. Cell viability was observed for 24 hours. This assay was conducted following the manufacturer's protocol. Briefly, 200 µL of 0.5 mg/mL 3-(4,5-dimethylthiazol-2-yl)-2,5-diphenyltetrazolium bromide (MTT; Thermo Fisher Scientific) solution was added to each well and incubated for 2 hours. Then, MTT was removed and washed with PBS twice per well. Dimethyl sulfoxide (DMSO; Merck) was added. After that, the optical density (OD) was determined at 570 nm by an Epoch microplate spectrophotometer (Biotek Instruments, Winooski, VT, United States). The percentage of cell viability was calculated as follows:



### Wound Healing Assay

The HGFs were seeded at a concentration of 1 × 10 ^5^ cells/well in 500 µL of DMEM containing 10% FBS in a 24-well plate and incubated at 5% CO _2_ and 37°C for 24 hours. After that, a single layer of HGFs was scraped with a P1000 pipette tip at the center of the well plate. The medium was removed and washed twice with PBS. HGFs were treated with 500 µL of PDLSC-CM at concentrations of 25 and 50 µg/mL. HGFs treated with DMEM with 10% FBS were used as controls. The plate was placed under an inverted microscope (Olympus CKX53, Shinjuku-ku, TYO Japan) using 4X magnification to take photos of the gap before the experiment. Cell migration was observed within 24 to 48 hours. Then, the medium was removed and washed with PBS twice per well. The HGFs were fixed in 100% methanol (Qrec, New Zealand) for 2 minutes before being rinsed twice with PBS. Then, 10% Giemsa stain (Merck) was added to stain HGFs for 10 minutes and rinsed with PBS twice. Photos were taken of the gap after the experiment. For each image, the HGFs that migrated to the created gap were counted by ImageJ software. The percentage of cell migration was calculated as follows:



### Boyden Chamber Assay

The HGFs were seeded in a 24-transwell microchemotaxis chamber at a concentration of 2.5 × 10 ^4^ cells/well/100 µL serum-free medium. A total of 750 µL of PDLSC-CM at concentrations of 25 and 50 µg/mL was applied to the lower chamber. DMEM with 10% FBS was used as a control. The HGFs were observed to induce cell chemotaxis within 24 hours. The medium in the upper chambers was removed, and two PBS washes per well were performed. The chambers were added to 100% methanol for 5 minutes and rinsed twice with PBS. The membranes were stained with 10% Giemsa stain for 15 minutes before being washed twice with PBS. The nonmigrated cells were removed with a cotton swab. The chambers were placed under the inverted microscope (Olympus CKX53) using 10X magnification to photograph cells moving across the membranes. The cells in each image were counted using ImageJ software. The percentage of cell migration was calculated as follows:



### Colorimetric Assay

The HGFs were seeded in a 24-well plate at a concentration of 5 × 10 ^3^ cells/well in 500 µL of DMEM with 10% FBS and incubated at 5% CO _2_ and 37°C for 24 hours. Next, the DMEM was removed, and the samples were washed twice with PBS. Then, 500 µL of PDLSC-CM at concentrations of 25 and 50 µg/mL was applied to treat HGFs. HGFs treated with DMEM containing 10% FBS were used as controls. Collagen synthesis was observed over 7 to 10 days. For collagen estimation, the medium was removed and washed twice with PBS. Then, 100% methanol was added for 10 minutes and rinsed with PBS twice per well. Then, the HGFs were stained with picrosirius red solution (Bio-optica, MI, Italy) according to the manufacturer's protocol. Photos were taken using 10X magnification under an inverted microscope (Nikon Eclipse TS100, Minato, TYO Japan). For quantification of collagen synthesis, collagen was dissolved in 0.1 M NaOH solution (VR Bioscience, BKK, Thailand) for 5 minutes. The OD was assessed at 540 nm using an Epoch microplate spectrophotometer. The percentage of collagen synthesis was calculated as follows:



### Gene Expression Analysis

For the evaluation of gene expression in the HGFs, real-time polymerase chain reaction (Realtime-PCR) was performed. The methodology for the treated HGFs was identical to the colorimetric assay. Collagen type I and type III gene expression was observed for 7 to 10 days. Total RNA was extracted with TRIzol reagent (Ambion, Austin, TX United States) according to the manufacturer's protocol. For each sample, 10 µL of RNA in molecular water was reverse transcribed to complementary DNA (cDNA) by reverse transcriptase supermix (Bio-Rad) according to the manufacturer's instructions. The sequences of specific primers for collagen type I ( *COL1A1* ) and type III ( *COL3A1* ) and the housekeeping gene ( *GAPDH* ) are shown in Table 1 . Realtime-PCR was performed using SYBR Green Supermix (Bio-Rad) according to the manufacturer's protocol. The result of Realtime-PCR was shown by increased fluorescent signals detected by the dye detector and measured as the C _T_ value. The relative gene expression levels were calculated by the 2 ^-∆∆CT^ method.

**Table 1:** Primer sequences used in real-time polymerase chain reaction

Gene		Sequence	Primer size (bp)	Melting temperature (Tm)	GenBank number
*COL1A1*	(F) (R)	5′-AACATGGAGACTGGTGAGACCT-3′ 5′-CGCCATACTCGAACTGGAATC-3′	145	60°C	NM_000088.4
*COL3A1*	(F) (R)	5′-AATGCCTGGAGAAAGAGGAGGT-3′ 5′-AATAGGACCAGTAGGACCCCTTG-3′	124	61°C	NM_000090.4
*GAPDH*	(F) (R)	5′-GGAGCGAGATCCCTCCAAAAT-3′ 5′-GGCTGTTGTCATACTTCTCATGG-3′	197	59°C	NM_001357943.2

Abbreviations: COL1A1, collagen type I; COL3A1, collagen type III; GAPDH, internal control.

### Statistical Analysis

The data were analyzed in terms of the mean ± standard deviation (SD). The Shapiro–Wilk test was used to determine whether the data had a normal distribution. All of the data were analyzed with one way analysis of variance (one-way ANOVA) and Bonferroni's post hoc test except cell viability assay, which was analyzed with Welch's test and Dunnett's T3 post hoc multiple comparison test. Statistical differences were calculated using SPSS version 18.0, with *p* < 0.05 consider significant ( *p* < 0.05).

## Results

### The Effect of PDLSC-CM on HGF Viability

**Fig. 1:**
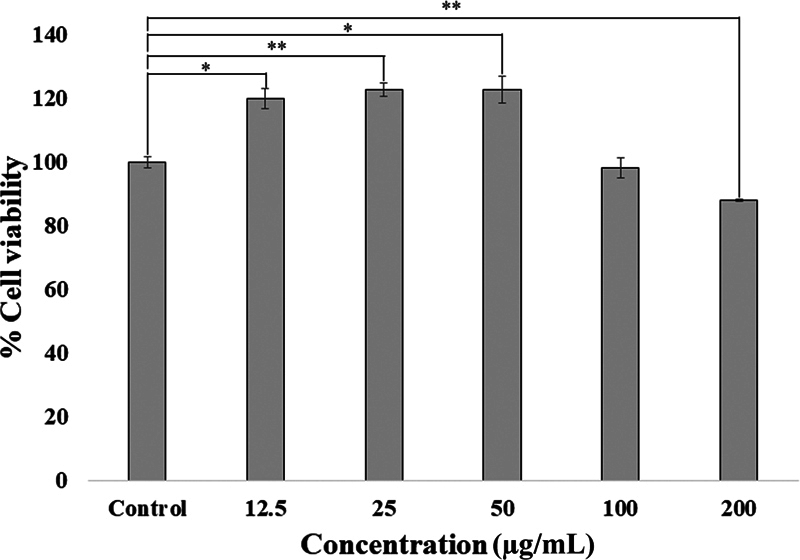
The percentage of cell viability of on human gingival fibroblasts (HGFs; mean ± SD; *n* = 3) on various concentrations (12.5–200 µg/mL) of periodontal ligament stem cell conditioned medium (PDLSC-CM). A significant difference is shown with an asterisk at * *p* < 0.05 and ** *p* < 0.001.

We examined the viability of HGFs treated with PDLSC-CM at various concentrations via the MTT assay. Fig. 1 depicts the percentage of HGF viability. The results showed that PDLSC-CM at 12.5, 25, and 50 µg/mL significantly increased the HGF viability to 119.98 ± 3.30% ( *p* < 0.05), 122.73 ± 2.11% ( *p* < 0.001), and 122.77 ± 4.10% ( *p* < 0.05), respectively, compared with the control (100 ± 1.63%). However, HGFs treated with 200 µg/mL PDLSC-CM showed a dramatically decreased cell viability to 88 ± 0.43% ( *p* < 0.001). Thus, PDLSC-CM at the best two concentrations, 25 and 50 µg/mL, was chosen to investigate the effect on HGF migration and collagen synthesis.

### The Enhancement of HGF Migration Using PDLSC-CM

**Fig. 2:**
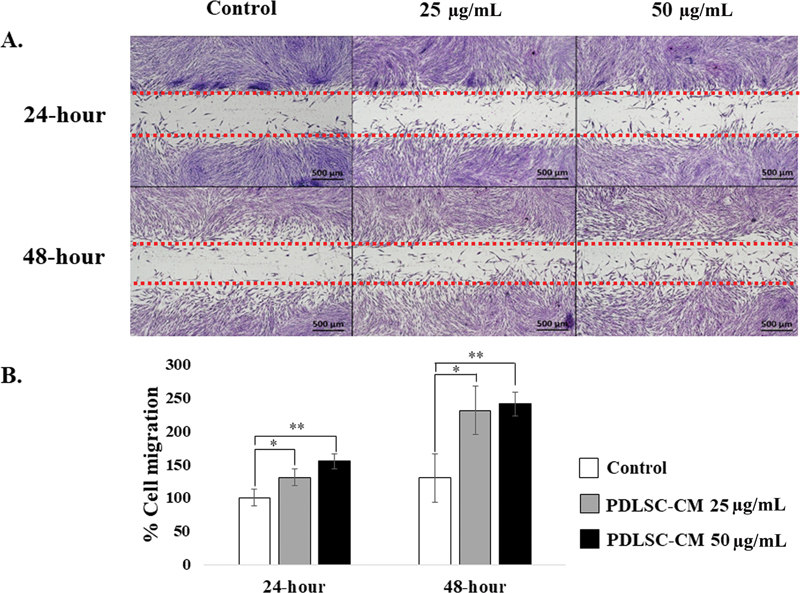
Wound healing assay. ( **A** ) The 4X magnification representative images of human gingival fibroblasts (HGFs) treated with control, 25 and 50 μg/mL of periodontal ligament stem cell conditioned medium (PDLSC-CM) at 24- and 48-hour time points as indicated. Scale bar = 500 μm. HGFs that migrated into the gaps were counted. ( **B** ) The percentage of HGF cell migration (mean ± SD, *n* = 3) on 25 and 50 µg/mL of PDLSC-CM are shown at 24- and 48-hour time points. A significant difference is shown with an asterisk at * *p* < 0.05 and ** *p* < 0.01.

To determine whether PDLSC-CM enhanced HGF cell migration, we used wound healing assay and Boyden chamber assay. The wound healing assay results are shown in Fig. 2 . At 24 hours post-seeding, the HGFs treated with 25 and 50 µg/mL PDLSC-CM showed a significant increase in the percentage of cell migration to 130.57 ± 12.61% ( *p* < 0.05) and 154.75 ± 11.41% ( *p* < 0.01), respectively, compared with the control (100 ± 12.71%). At 48 hours post-seeding, the percentage of cell migration was significantly increased to 230.74 ± 36.64% ( *p* < 0.05) and 240.41 ± 17.95% ( *p* < 0.01) when HGFs were treated with 25 and 50 µg/mL PDLSC-CM, respectively, compared with the control (129.88 ± 36.39%).

**Fig. 3:**
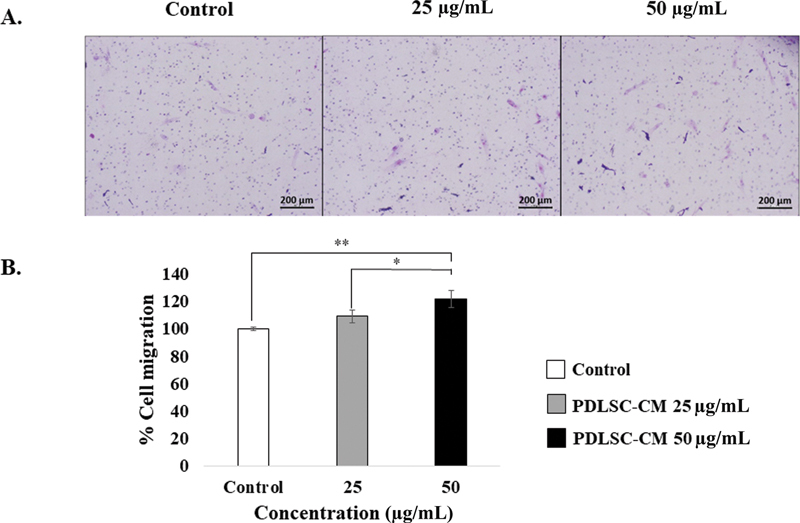
Boyden chamber assay. ( **A** ) The 10X magnification representative images of human gingival fibroblasts (HGFs) treated with control, 25 and 50 μg/mL of periodontal ligament stem cell conditioned medium (PDLSC-CM) at 24-hour time points as indicated. Scale bar = 200 μm. The migrated cells were counted. ( **B** ) The percentages of HGF cell migration (mean ± SD, *n* = 3) on 25 and 50 μg/mL of PDLSC-CM are shown at 24-hour time points. A significant difference is shown with an asterisk at * *p* < 0.05 and ** *p* < 0.01.

Next, we examined the effect of the chemotactic stimulus PDLSC-CM on HGF migration. Fig. 3 illustrates the Boyden chamber assay outcomes. At the 24-hour observational time point, 50 µg/mL PDLSC-CM significantly enhanced the HGF migration rate to 126.83 ± 10.07% ( *p* < 0.01) when compared with the control (100 ± 1.06%). Nevertheless, 25 µg/mL PDLSC-CM did not increase the HGF migration rate at this time point.

### PDLSC-CM Promoted Collagen Secretion from HGFs

**Fig. 4:**
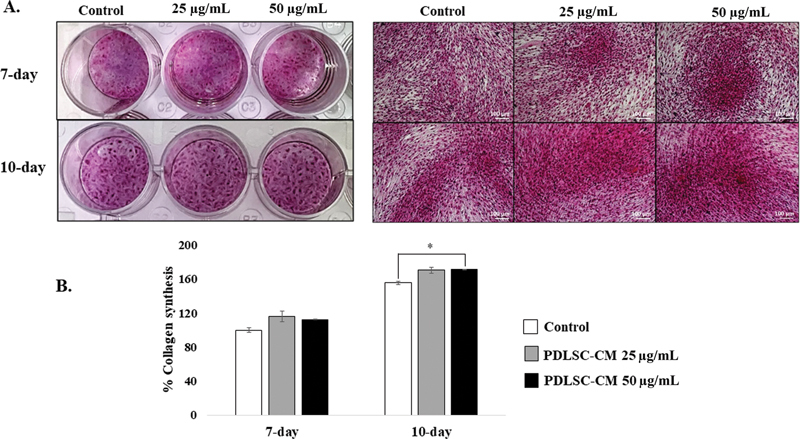
Colorimetric assay. ( **A** ) The images of the picrosirius red staining and the 10X magnification representative images of human gingival fibroblasts (HGFs) treated with the control group and the 25 and 50 µg/mL periodontal ligament stem cell conditioned medium (PDLSC-CM) groups at 7- and 10-day time points as indicated. Scale bar = 100 μm. The picrosirius red staining was dissolved. ( **B** ) The percentage of collagen synthesis from HGF cells (mean ± SD, *n* = 3) on 25 and 50 μg/mL of PDLSC-CM are shown at 7- and 10-day time points. A significant difference is shown with an asterisk at * *p* < 0.05.

Collagen secretion is an important function of HGFs during wound healing. The amount of collagen was evaluated using a colorimetric assay and gene expression analysis. Fig. 4A shows collagen staining in the control group and the 25 and 50 µg/mL PDLSC-CM groups at 7 and 10 days, as indicated. After that, the picrosirius red staining was eluted from the wells. Fig. 4B displays the percentage of collagen synthesis determined via the colorimetric assay. At the 7-day observational time point, neither (25 and 50 µg/mL) of the PDLSC-CMs induced the HGFs to secrete collagen at higher levels than the control. In contrast, 50 µg/mL PDLSC-CM significantly increased the percentage of collagen synthesis to 163.21 ± 4.38% ( *p* < 0.05) compared with the control (149.88 ± 1.36%) at the 10-day observational time point. Nonetheless, the amount of collagen production was not different between the 25 µg/mL PDLSC-CM group and the control group at 10 days post-seeding.

### Upregulation of Collagen Type I and III Synthesis from HGFs via PDLSC-CM

**Fig. 5:**
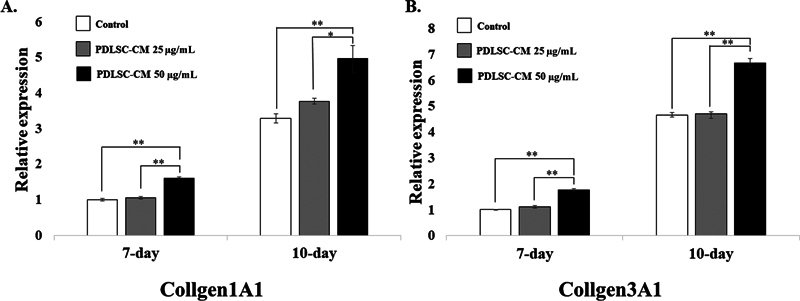
Gene expression analysis. Chart of ( **A** ) *COL1A1* gene expression at 7- and 10-day time points and ( **B** ) *COL3A1* gene expression at 7- and 10-day time points. The relative normalized expression of human gingival fibroblasts (HGFs; mean ± SD, *n* = 3) on 25 and 50 µg/mL of periodontal ligament stem cell conditioned medium (PDLSC-CM) was calculated. A significant difference is shown with an asterisk at * *p* < 0.01 and ** *p* < 0.001.

Since the HGFs normally secrete collagen types I and III during the early wound healing process, we particularly focused on *COL1A1* and *COL3A1* gene expression. HGFs treated with 50 µg/mL PDLSC-CM resulted in significantly higher *COL1A1* expression (1.6-fold [ *p* < 0.001] and 4.96-fold [ *p* < 0.001]) than the control at 7 and 10 days post-seeding, respectively ( Fig. 5 ). Similarly, at this concentration, *COL3A1* gene expression was significantly induced in the HGFs from 1.76-fold ( *p* < 0.001) and 6.67-fold ( *p* < 0.001) compared with that of the control at 7 and 10 days after seeding, respectively. However, there was no difference between the 25 µg/mL PDLSC-CM group and the control group in *COL1A1* and *COL3A1* gene expression at any time points.

## Discussion

This study aimed to explore the effectiveness of PDLSC-CM in promoting HGF migration and collagen synthesis. We demonstrated that PDLSC-CM at 50 µg/mL concentration enhanced early periodontal wound healing by stimulating HGF migration and collagen production. According to Lin and colleagues, there are three proposed mechanisms for how MSC-CM stimulates periodontal regeneration. First, MSC-CM, which contains VEGF and fibroblast growth factor-2 (FGF-2), enhances early angiogenesis by increasing endothelial cell proliferation and migration. Second, MSC-CM improved immunomodulatory properties by upregulating the anti-inflammatory cytokines and downregulating the proinflammatory cytokines. Finally, MSC-CM enhances macrophages, osteogenic precursor cells, and angiogenic precursor cell chemotaxis to the wound site. 21 A previous study showed that PDLSC-CM at moderate to high concentrations promoted periodontal regeneration. This study did not provide exact concentrations. 7 Nevertheless, another study found that PDLSC-CM at a low concentration (<70 µg/mL) enhances HGF viability and proliferation. 22 Aghamohamadi and colleagues revealed that concentrations higher and lower than 6.25 mg/ml of PDLSC-CM inhibited the PDLSC proliferation rate. Thus, PDLSC-CM concentrations influenced our results as well as the quality of wound healing. 23

In this study, we anticipated that different PDLSC-CM concentrations would affect cell viability. Daskalaki and colleagues used liquid chromatography high-resolution mass spectrometry to detect cell metabolites under different conditions. The results showed that PDLSC-CM at a low concentration (<100 µg/mL) substantially increased the viability of HGFs. 24 Likewise, Jantanasan and colleagues investigated whether PDLSC-CM concentrations of 8.75 to 35 µg/mL enhanced HGF viability. These concentrations also promoted cell proliferation. Furthermore, compared with the stem cells from human exfoliated deciduous tooth conditioned medium (SHED-CM), PDLSC-CM showed a better potential to induce cell proliferation. 22 Nonetheless, when the concentration of PDLSC-CM was elevated to 200 µg/mL, HGF viability declined. This finding revealed that distinct proteins and other cell metabolites would emerge depending on the medium combinations. 24 Growth inhibitors, such as ammonium and lactate, accumulate in cell culture. 25 For this reason, different concentrations and types of medium affect HGF viability.

Cell migration assays are also used for wound healing. We hypothesized that PDLSC-CM could improve HGF migration. The wound healing assay results show that both PDLSC-CM concentrations (25 and 50 µg/mL) enhanced HGF migration. However, only a 50 µg/mL concentration was found to stimulate cell migration using the Boyden chamber assay. The stem cell secretome contains a variety of growth factors as mentioned earlier. Nishimura and Terranova performed experiments on the responsiveness of the HGFs to polypeptide growth factors. The migratory rate of HGFs treated with PDGF, insulinlike growth factor-I (IGF-I), IGF-II, epidermal growth factor (EGF), and transforming growth factor β (TGF-β) was elevated. 26 For this reason, PDLSC-CM increased the HGF migration rate, resulting in expedited wound closure. From the laboratory methodology, the Boyden chamber assay was evaluated only at the 24-hour time point. According to the pivot study, our results at the 48-hour time point were impacted by the proliferation of HGFs. To assess HGF migration, we used the wound healing assay and the Boyden chamber assay in this study. The wound healing assay or scratch assay is used for studying intracellular events by mimicking cell migration in vivo by creating a gap between the cells. 27 28 The Boyden chamber assay is especially well suited for analyzing cell migration as a consequence of various stimuli, such as growth factors. 29

Finally, collagen plays an essential role in the proliferative and remodeling phases of wound healing. Collagen type III is secreted by the HGFs for wound contraction, and collagen type I is generated for wound repair in the late phase of wound healing. Clark described wound repair in which collagen deposition begins ∼3 days after injury or following granulation tissue formation. Complete collagen remodeling requires several months. 30 According to this study, 50 µg/mL PDLSC-CM increased *COL1A1* and *COL3A1* gene expression at 10 days after seeding. This concentration enhanced collagen synthesis, as shown by the picrosirius red staining. *COL1A1* and *COL3A1* gene expression demonstrated a higher level in 50 µg/mL PDLSC-CM at 7 days post-seeding despite the fact that colorimetric assay would be unable to identify collagen synthesis between the test and control groups. Picrosirius red is not a highly sensitive method for detecting collagen synthesis, especially when collagen is negligible. 31 Additionally, picrosirius red is not specific to collagen due to sulfonic group in the picrosirius red solution reacting with basic amino acids of collagen including hydroxyproline and proline. 32 These amino acid groups can be found in various cell structures, for example, basal lamina. These reasons explain why the collagen synthesis in the test groups was not significantly different from the control in colorimetric assay at a 7-day time point. Collagen synthesis was confirmed at the mRNA level using Realtime-PCR. The results of this study showed that PDLSC-CM promoted collagen synthesis in the early phase of wound healing, indicating rapid wound contraction and wound repair.

Applications of CM have been widely applied to treat degenerative diseases, 33 cerebral ischemia, 34 muscle atrophy, 35 36 and bony defects. 37 38 Benavides-Castellanos and colleagues performed a systematic review and meta-analysis of the effect of MSC-CM on bone regeneration. MSC-CM enhances bone formation in the bone fracture model by promoting angiogenesis and osteogenesis. 37 In a human case report, it was found that MSC-CM increases bone mineralization in the lateral window sinus floor elevation procedure. 38 Additionally, CM demonstrates an improvement of the delayed wound healing process in immunocompromised hosts. Saheli and colleagues investigated whether MSC-CM could improve diabetic wound healing in a rat model. They reported that under diabetic conditions, MSC-CM suppresses inflammatory responses and increases fibroblasts viability, proliferation, and collagen synthesis. 39 Utilizing CM for periodontal defects is intriguing, given the many advantages. Considering the CM vehicle, β-tricalcium phosphate or atelocollagen sponge is immersed in the CM. 7 38 The combination of propylene glycol alginate and an enamel matrix derivative enhances periodontal regeneration. 40 Thus, the CM vehicle is important for further development and preparation.

The primary mediator of the therapeutic effects of PDLSC-CM on HGF viability, migration, and collagen synthesis is an intriguing topic for discussion. Growth factors and cytokines may have an impact on HGF functions. Combining growth factors, including PDGF, TGF-β, vascular endothelial growth factor (VEGF), and IGF, has been proven to accelerate cell proliferation and migration and promote gingival regeneration. 41 Besides, interleukin-1β (IL-1β), IL-6, tumor necrosis factor-α (TNF-α), PDGF, and TGF-β stimulate HGF functions and enhance HGFs to generate extracellular matrix. 42 MSC-EVs improve angiogenesis, osteoblast proliferation, differentiation, intercellular crosstalk, and balance innate and adaptive immunity to increase bone regeneration. 11 MSC-EVs additionally aid in periodontal regeneration by the formation of periodontium and suppression of the inflammatory response. 43 44 Thus, PDLSC secretome, including growth factors, cytokines, and EVs, may get involved on HGF viability, migration, and collagen synthesis. To the best of our knowledge, this is the first study to investigate the effects of PDLSC-CM on HGFs. It would be fascinating to discover more about the principal agents in PDLSC-CM in future research.

## Conclusion

We demonstrated that PDLSC-CM at a concentration of 50 µg/mL increased HGF viability, migration, and collagen synthesis in vitro. Thus, this study suggested that PDLSC-CM enhanced rapid wound closure and optimized the quality of early wound healing, particularly in periodontal defects. According to our research's limitations, adding FBS to the test group cannot be avoid. FBS contains several growth factors required for cell proliferation and cell attachment. Therefore, FBS may influence the outcomes. For collagen estimation, HGFs were cultured for 7 to 10 days. The proliferation of HGFs may be of concern in this period. Likewise, picrosirius red is not particularly sensitive or specific to collagen, as has already been mentioned. Further research is necessary to confirm the effect of PDLSC-CM on additional protein analysis and in vivo may be performed.
